# Supplementary material for: Effects of 3-Hydroxy-3-methylglutaryl-CoA Reductase Inhibitors on Cholesterol Metabolism in Laying Hens
Source: Animals (Basel). 2023 Jun 3;13(11):1868. doi: 10.3390/ani13111868 (PMC10251945; doi:10.3390/ani13111868)
Supplement: Supplementary file 1 [file animals-13-01868-s001.zip › animals-2396302-supplementary.pdf]

Supplementary Table S1. Composition of basal diets and nutrient levels.

Ingredients	Percentage (%)	Nutrient level	Content
Wheat	68.40	ME (kcal/kg)	2750
Soybean meal	15.00	Crude protein (%)	16.50
Soybean oil	1.30	Calcium (%)	3.60
DDGS	3.00	Available phosphorus (%)	0.35
Limestone	9.00	Met (%)	0.35
$CaHPO_4$	0.80	Lys (%)	0.80
Bran	1.00	Met + Cys (%)	0.70
Premix[1]	1.50		

[1]The premix provided the following per kg of diet: vitamin A, 15,000 IU; cholecalciferol 4,000 IU; vitamin E, 50 mg; thiamine, 3.60 mg; riboflavin, 10 mg; pyridoxine, 5.5 mg; iron, 100 mg; manganese, 110 mg; copper, 10 mg; zinc, 100 mg; selenium, 0.4 mg; iodine, 0.3 mg.

Supplementary Table S2. Primers for PCR[1].

Gene name	Accession	Primer sequence (5'-3')	Product size (bp)	Amplification efficiency (%)
HMGCR	NM_204485.3	F: AGGAGCTTGCTGTGAAAACG<br>R: AAGCTTTCACTTCTGCAGCC	254	99.7
MVK	XM_015275510.4	F: TCGGGTGTGGATAATGCTGT<br>R: CCAGAACACTTTGGCACTCC	238	98.6
FDPS	NM_001396601.1	F: AGGAGTTCGTGGGGTTCTTC<br>R: AACAACTCGATGCACCAACC	241	98.3
FDFT1	NM_001039294.2	F: AAGGTCCCGATGCTGAATGA<br>R: AGAGAAGAGTCGGGAAAGGC	288	99.8
SQLE	NM_001194927.2	F: TTGTGGGTTCAGGTGTCCTT<br>R: CCTCCGACTTGCTCTCTAGG	234	99.5
VLDLR	NM_205229.2	F: AAGGACTGGAGTGATGAGCC<br>R: GTTCTTTCCCCACAGCCTTG	289	99.2
β-actin	NM_205518.2	F: AGTACCCCATTGAACACGGT<br>R: ATACATGGCTGGGGTGTTGA	197	98.5

[1] Abbreviation represents: *HMGCR*, 3-hydroxy-3-methylglutaryl-CoA reductase; *MVK*, mevalonate kinase; *FDPS*, farnesyl diphosphate synthase; *FDFT1*, farnesyl-diphosphate farnesyltransferase 1; *SQLE*, squalene epoxidase; *VLDLR*, very low density lipoprotein receptor; *β-actin*, actin beta.
